# A second monoclinic polymorph of 2,4-dimethyl­anilinium chloride

**DOI:** 10.1107/S160053681104551X

**Published:** 2011-11-05

**Authors:** Soudeh Hossein Zadeh, Mohamad Reza Talei Bavil Olyai, Hossein Biglari Mazlaghani, Behrouz Notash

**Affiliations:** a Department of Chemistry, Islamic Azad University, Karaj Branch, Karaj, Iran; b Department of Chemistry, Islamic Azad University, South Tehran Branch, Tehran, Iran; c Department of Chemistry, Shahid Beheshti University, G. C., Evin, Tehran 1983963113, Iran

## Abstract

A second monoclinic polymorph of 2,4-dimethyl­anilinium chloride, C_8_H_12_N^+^·Cl^−^, (I), is reported. The unit-cell dimensions differ from those of the first reported monoclinic form, (II) [Yao (2010 ▶). *Acta Cryst.* E**66**, o1563]. Nevertheless, both compounds crystallize in the monoclinic space group *P*2_1_/*n*. As in (II), the protonated amine group in (I) acts as a hydrogen-bond donor to the chloride ion, forming three N—H⋯Cl hydrogen bonds. The result is a two-dimensional network in the *ac* plane. The difference in the hydrogen-bond pattern is that in (I) only 12-membered rings are formed whereas in (II), eight-membered and 16-membered rings are formed.

## Related literature

For another monoclinic polymorph of the title compound, see: Yao (2010 ▶). For properties of compounds containing inorganic anions and organic cations, see: Masse *et al.* (1993 ▶); Xiao *et al.* (2005 ▶).
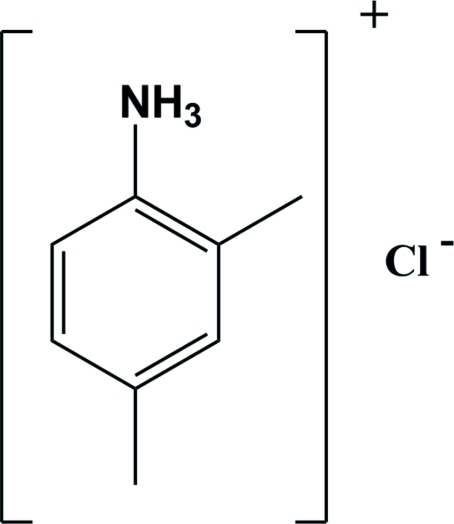


## Experimental

### 

#### Crystal data

C_8_H_12_N^+^·Cl^−^
*M* *_r_* = 157.64
Monoclinic, 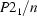

*a* = 5.3651 (11) Å
*b* = 18.631 (4) Å
*c* = 8.5428 (17) Å
β = 94.48 (3)°
*V* = 851.3 (3) Å^3^
*Z* = 4
Mo *K*α radiation
μ = 0.38 mm^−1^
*T* = 298 K
0.45 × 0.4 × 0.34 mm

#### Data collection

Stoe IPDS 2T diffractometer
5557 measured reflections
2276 independent reflections
1838 reflections with *I* > 2σ(*I*)
*R*_int_ = 0.031

#### Refinement

*R*[*F* ^2^ > 2σ(*F* ^2^)] = 0.042
*wR*(*F* ^2^) = 0.104
*S* = 1.09
2276 reflections
105 parameters
H atoms treated by a mixture of independent and constrained refinement
Δρ_max_ = 0.22 e Å^−3^
Δρ_min_ = −0.17 e Å^−3^

### 

Data collection: *X-AREA* (Stoe & Cie, 2005 ▶); cell refinement: *X-AREA*; data reduction: *X-AREA*; program(s) used to solve structure: *SHELXS97* (Sheldrick, 2008 ▶); program(s) used to refine structure: *SHELXL97* (Sheldrick, 2008 ▶); molecular graphics: *ORTEP-3 for Windows* (Farrugia, 1997 ▶); software used to prepare material for publication: *WinGX* (Farrugia, 1999 ▶).

## Supplementary Material

Crystal structure: contains datablock(s) I, global. DOI: 10.1107/S160053681104551X/bt5679sup1.cif

Structure factors: contains datablock(s) I. DOI: 10.1107/S160053681104551X/bt5679Isup2.hkl

Supplementary material file. DOI: 10.1107/S160053681104551X/bt5679Isup3.cml

Additional supplementary materials:  crystallographic information; 3D view; checkCIF report

## Figures and Tables

**Table 1:** Hydrogen-bond geometry (Å, °)

*D*—H⋯*A*	*D*—H	H⋯*A*	*D*⋯*A*	*D*—H⋯*A*
N1—H1*A*⋯Cl1^i^	0.91 (2)	2.28 (2)	3.1721 (17)	164.4 (17)
N1—H1*B*⋯Cl1^ii^	0.88 (2)	2.35 (2)	3.2228 (17)	172.1 (18)
N1—H1*C*⋯Cl1^iii^	0.94 (2)	2.23 (2)	3.1630 (16)	172.8 (19)

Symmetry codes: (i) 
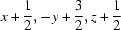
; (ii) 
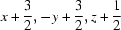
; (iii) 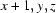.

**Fig. 1.**
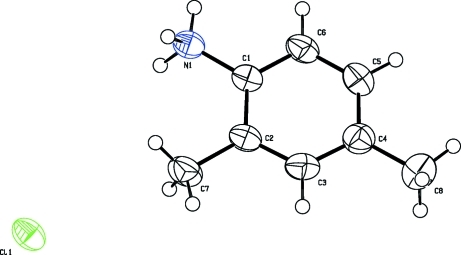
The molecular structure of the title compound with the atom-numbering scheme. Displacement ellipsoids are drawn at the 50% probability level.

**Fig. 2.**
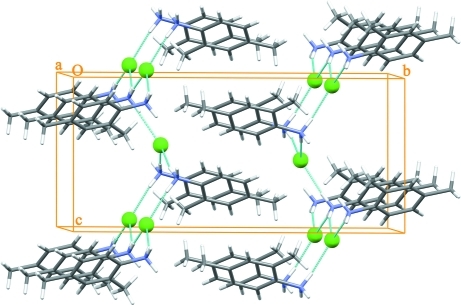
Packing diagram of the title compound showing intermolecular N—H···Cl hydrogen bonding.

## supplementary crystallographic information

### Comment

The combination of organic cations and inorganic anions is important in material science and chemistry because of their abilities to join the properties of organic and inorganic molecules,  exhibiting some interesting crystal structures with special properties, such as luminescence, magnetism, and multifunctional properties (Masse *et al.*, 1993). Furthermore these hybrid materials have a great interest due to their numerous varieties of intriguing structural topologies (Xiao *et al.*, 2005).

The title compound, (I), is a second monoclinic polymorph of the structure of 2,4-dimethylanilinium chloride which crystallizes in the space group *P*2_1_/*n*. A different structure, (II), in the space group *P*2_1_/*c* was described earlier by (Yao, 2010).

The asymmetric unit of the title compound contains one 2,4-dimethylanilinium cation and one chloride anion (Fig. 1). The cell parameters of the current monoclinic polymorph vary significantly from the earlier polymorph [*a* = 9.4739 (19) Å, *b* = 9.894 (2) Å, *c* = 9.6709 (19) Å and *β* = 96.31 (3)°]. The C—N bond length is 1.465 (2) Å. The C—N bond length for previous crystal structure was given 1.466 (3) Å (Yao, 2010). Inside lattice, each Cl^-^ anion is linked to three organic species through N–H···Cl hydrogen bonds  (Table 1).

The two-dimensional arrangement of the chloride anions and 2,4-dimethylanilinum cations in the unit cell is shown in Fig. 2.

### Experimental

An ethanolic solution of 2,4-dimethylaniline (1.21 g, 10 mmol in 10 ml) was added dropwise to a magnetically stirred of aqueous hydrochloric acid solution (1.01 g, 10 mmol) a 1:1 molar ratio. The achieved solution is then filtered to eliminate the colorless crystals precipitated formed and then stirred for 2 hrs. After stirring, the obtained solution was slowly evaporated at room temperature over several days resulting in the formation of 2,4-dimethylanilinium chloride crystals.

### Refinement

The hydrogen atoms of the protonated nitrogen were found in difference Fourier map and refined isotropically. The C—H protons were positioned geometrically and refined as riding atoms with C—H = 0.93 Å and *U*iso(H) = 1.2 *U*eq(C) for aromatic C—H  and C—H = 0.96 Å and *U*iso(H) = 1.5 *Ueq*(C) for methyl groups.

### Crystal data

**Table:**

C_8_H_12_N^+^·Cl^−^	*F*(000) = 336
*M**_r_* = 157.64	*D*_x_ = 1.230 Mg m^−^^3^
Monoclinic, *P*2_1_/*n*	Mo *K*α radiation, λ = 0.71073 Å
Hall symbol: -P 2yn	Cell parameters from 2276 reflections
*a* = 5.3651 (11) Å	θ = 2.2–29.1°
*b* = 18.631 (4) Å	µ = 0.38 mm^−^^1^
*c* = 8.5428 (17) Å	*T* = 298 K
β = 94.48 (3)°	Block, colorless
*V* = 851.3 (3) Å^3^	0.45 × 0.4 × 0.34 mm
*Z* = 4	

### Data collection

**Table:**

Stoe IPDS 2T diffractometer	1838 reflections with *I* > 2σ(*I*)
Radiation source: fine-focus sealed tube	*R*_int_ = 0.031
graphite	θ_max_ = 29.1°, θ_min_ = 2.2°
Detector resolution: 0.15 mm pixels mm^-1^	*h* = −7→6
rotation method scans	*k* = −22→25
5557 measured reflections	*l* = −11→11
2276 independent reflections	

### Refinement

**Table:**

Refinement on *F*^2^	Primary atom site location: structure-invariant direct methods
Least-squares matrix: full	Secondary atom site location: difference Fourier map
*R*[*F*^2^ > 2σ(*F*^2^)] = 0.042	Hydrogen site location: inferred from neighbouring sites
*wR*(*F*^2^) = 0.104	H atoms treated by a mixture of independent and constrained refinement
*S* = 1.09	*w* = 1/[σ^2^(*F*_o_^2^) + (0.0462*P*)^2^ + 0.1562*P*] where *P* = (*F*_o_^2^ + 2*F*_c_^2^)/3
2276 reflections	(Δ/σ)_max_ < 0.001
105 parameters	Δρ_max_ = 0.22 e Å^−^^3^
0 restraints	Δρ_min_ = −0.17 e Å^−^^3^

### Special details

**Table:**

Geometry. All e.s.d.'s (except the e.s.d. in the dihedral angle between two l.s. planes) are estimated using the full covariance matrix. The cell e.s.d.'s are taken into account individually in the estimation of e.s.d.'s in distances, angles and torsion angles; correlations between e.s.d.'s in cell parameters are only used when they are defined by crystal symmetry. An approximate (isotropic) treatment of cell e.s.d.'s is used for estimating e.s.d.'s involving l.s. planes.
Refinement. Refinement of *F*^2^ against ALL reflections. The weighted *R*-factor *wR* and goodness of fit *S* are based on *F*^2^, conventional *R*-factors *R* are based on *F*, with *F* set to zero for negative *F*^2^. The threshold expression of *F*^2^ > σ(*F*^2^) is used only for calculating *R*-factors(gt) *etc*. and is not relevant to the choice of reflections for refinement. *R*-factors based on *F*^2^ are statistically about twice as large as those based on *F*, and *R*- factors based on ALL data will be even larger.

### Fractional atomic coordinates and isotropic or equivalent isotropic displacement parameters (Å^2^)

**Table:**

	*x*	*y*	*z*	*U*_iso_*/*U*_eq_	
C8	0.9245 (5)	0.39088 (11)	0.1976 (3)	0.0692 (6)	
H8A	1.0616	0.3635	0.2452	0.104*	
H8B	0.9148	0.3842	0.0858	0.104*	
H8C	0.7715	0.3750	0.2376	0.104*	
C7	0.6327 (3)	0.64679 (10)	0.1382 (2)	0.0533 (4)	
H7A	0.6040	0.6834	0.2139	0.080*	
H7B	0.4783	0.6227	0.1078	0.080*	
H7C	0.6969	0.6683	0.0475	0.080*	
Cl1	0.08615 (7)	0.78443 (2)	0.04928 (5)	0.04796 (14)	
N1	1.0561 (3)	0.68899 (8)	0.35343 (16)	0.0406 (3)	
C1	1.0245 (3)	0.61337 (8)	0.30934 (16)	0.0367 (3)	
C2	0.8198 (3)	0.59328 (9)	0.20976 (17)	0.0407 (3)	
C3	0.7952 (3)	0.52034 (10)	0.17611 (19)	0.0477 (4)	
H3	0.6585	0.5053	0.1107	0.057*	
C5	1.1683 (3)	0.49210 (10)	0.3321 (2)	0.0502 (4)	
H5	1.2861	0.4589	0.3719	0.060*	
C4	0.9645 (3)	0.46926 (9)	0.23530 (19)	0.0478 (4)	
C6	1.1980 (3)	0.56416 (9)	0.37005 (19)	0.0458 (4)	
H6	1.3341	0.5792	0.4360	0.055*	
H1A	0.932 (4)	0.7056 (11)	0.411 (3)	0.057 (6)*	
H1C	1.060 (4)	0.7208 (11)	0.268 (3)	0.062 (6)*	
H1B	1.192 (5)	0.6971 (11)	0.415 (2)	0.057 (6)*	

### Atomic displacement parameters (Å^2^)

**Table:**

	*U*^11^	*U*^22^	*U*^33^	*U*^12^	*U*^13^	*U*^23^
C8	0.0840 (16)	0.0542 (12)	0.0681 (12)	0.0007 (11)	−0.0025 (11)	−0.0061 (9)
C7	0.0395 (9)	0.0614 (11)	0.0569 (10)	0.0008 (8)	−0.0108 (7)	0.0054 (8)
Cl1	0.0356 (2)	0.0578 (3)	0.0501 (2)	−0.00034 (18)	0.00128 (14)	0.01375 (18)
N1	0.0323 (7)	0.0494 (8)	0.0397 (7)	−0.0023 (6)	0.0003 (5)	0.0018 (6)
C1	0.0299 (7)	0.0462 (8)	0.0342 (6)	−0.0031 (6)	0.0036 (5)	0.0034 (6)
C2	0.0325 (7)	0.0526 (9)	0.0366 (7)	−0.0016 (7)	0.0001 (5)	0.0035 (6)
C3	0.0437 (9)	0.0567 (10)	0.0415 (8)	−0.0067 (7)	−0.0051 (6)	−0.0019 (7)
C5	0.0442 (9)	0.0532 (10)	0.0525 (9)	0.0065 (8)	−0.0007 (7)	0.0080 (7)
C4	0.0530 (10)	0.0499 (9)	0.0406 (8)	−0.0012 (8)	0.0048 (7)	0.0010 (7)
C6	0.0345 (8)	0.0553 (10)	0.0462 (8)	−0.0017 (7)	−0.0056 (6)	0.0062 (7)

### Geometric parameters (Å, °)

**Table:**

C8—C4	1.507 (3)	N1—H1B	0.88 (2)
C8—H8A	0.9600	C1—C6	1.379 (2)
C8—H8B	0.9600	C1—C2	1.387 (2)
C8—H8C	0.9600	C2—C3	1.393 (2)
C7—C2	1.509 (2)	C3—C4	1.383 (2)
C7—H7A	0.9600	C3—H3	0.9300
C7—H7B	0.9600	C5—C4	1.385 (3)
C7—H7C	0.9600	C5—C6	1.387 (3)
N1—C1	1.465 (2)	C5—H5	0.9300
N1—H1A	0.91 (2)	C6—H6	0.9300
N1—H1C	0.94 (2)		
			
C4—C8—H8A	109.5	C6—C1—C2	122.02 (15)
C4—C8—H8B	109.5	C6—C1—N1	118.79 (14)
H8A—C8—H8B	109.5	C2—C1—N1	119.18 (14)
C4—C8—H8C	109.5	C1—C2—C3	116.57 (15)
H8A—C8—H8C	109.5	C1—C2—C7	122.66 (15)
H8B—C8—H8C	109.5	C3—C2—C7	120.76 (15)
C2—C7—H7A	109.5	C4—C3—C2	123.15 (16)
C2—C7—H7B	109.5	C4—C3—H3	118.4
H7A—C7—H7B	109.5	C2—C3—H3	118.4
C2—C7—H7C	109.5	C4—C5—C6	120.51 (16)
H7A—C7—H7C	109.5	C4—C5—H5	119.7
H7B—C7—H7C	109.5	C6—C5—H5	119.7
C1—N1—H1A	112.9 (13)	C3—C4—C5	118.14 (16)
C1—N1—H1C	114.4 (12)	C3—C4—C8	120.66 (17)
H1A—N1—H1C	105.4 (18)	C5—C4—C8	121.19 (18)
C1—N1—H1B	113.2 (13)	C1—C6—C5	119.59 (15)
H1A—N1—H1B	103.1 (18)	C1—C6—H6	120.2
H1C—N1—H1B	106.9 (18)	C5—C6—H6	120.2
			
C6—C1—C2—C3	1.2 (2)	C2—C3—C4—C8	178.59 (17)
N1—C1—C2—C3	−177.97 (14)	C6—C5—C4—C3	1.1 (2)
C6—C1—C2—C7	−178.00 (15)	C6—C5—C4—C8	−177.79 (17)
N1—C1—C2—C7	2.9 (2)	C2—C1—C6—C5	−0.4 (2)
C1—C2—C3—C4	−0.8 (2)	N1—C1—C6—C5	178.72 (15)
C7—C2—C3—C4	178.37 (15)	C4—C5—C6—C1	−0.7 (3)
C2—C3—C4—C5	−0.3 (2)		

### Hydrogen-bond geometry (Å, °)

**Table:**

*D*—H···*A*	*D*—H	H···*A*	*D*···*A*	*D*—H···*A*
N1—H1A···Cl1^i^	0.91 (2)	2.28 (2)	3.1721 (17)	164.4 (17)
N1—H1B···Cl1^ii^	0.88 (2)	2.35 (2)	3.2228 (17)	172.1 (18)
N1—H1C···Cl1^iii^	0.94 (2)	2.23 (2)	3.1630 (16)	172.8 (19)

Symmetry codes: (i) *x*+1/2, −*y*+3/2, *z*+1/2; (ii) *x*+3/2, −*y*+3/2, *z*+1/2; (iii) *x*+1, *y*, *z*.
